# Transcriptional response of *Meloidogyne incognita* to non-fumigant nematicides

**DOI:** 10.1038/s41598-022-13815-9

**Published:** 2022-06-13

**Authors:** Catherine L. Wram, Cedar N. Hesse, Inga A. Zasada

**Affiliations:** 1 Department of Botany and Plant Pathology, Oregon State University, Corvallis, OR 97331 USA; 2 USDA-ARS Horticultural Crops Research Unit, Corvallis, OR 97330 USA

**Keywords:** Agricultural genetics, Gene expression, Toxicology, Transcriptomics

## Abstract

There is limited research about the impacts of new nematicides, including fluazaindolizine, fluopyram, and fluensulfone, on the plant-parasitic nematode *Meloidogyne incognita*, despite it being a pervasive agricultural pest. In this study, *M. incognita* second-stage juveniles were exposed for 24-h to fluensulfone, fluazaindolizine, fluopyram, and oxamyl and total RNA was extracted and sequenced using next-generation sequencing to determine gene expression. The effects of nematicide exposure on cellular detoxification pathways, common differentially expressed (DE) genes, and fatty acid and retinol-binding genes were examined. Fluopyram and oxamyl had the smallest impacts on the *M. incognita* transcriptome with 48 and 151 genes that were DE, respectively. These compounds also elicited a weak response in the cellular detoxification pathway and fatty acid and retinol-binding (FAR) genes. Fluensulfone and fluazaindolizine produced robust transcriptional responses with 1208 and 2611 DE genes, respectively. These compounds had strong impacts on cellular detoxification, causing differential regulation of transcription factors and genes in the detox pathway. These compounds strongly down-regulated FAR genes between 52–85%. Having a greater understanding of how these compounds function at a molecular level will help to promote proper stewardship, aid with nematicide discovery, and help to stay a step ahead of nematicide resistance.

## Introduction

Of the plant-parasitic nematodes (PPN) that cause over $118 billion in damage and crop loss globally each year, species in the genus *Meloidogyne* are among the most devastating^1,2^. *Meloidogyne incognita* (southern root-knot nematode) is the most prevalent and destructive of these species because its widespread global distribution and ability to infect a broad range of agriculturally important crops^3–5^. In the United States, *M. incognita* can be found in 29 states^6^. Management of PPN has relied on fumigant nematicides (methyl bromide, chloropicrin, and 1,3-dichloropropene) because of their broad-spectrum activity on weeds, fungi and nematodes. However, these compounds also have adverse effects both on human health and the environment, and therefore, have been gradually phased out or more heavily regulated over the last 30 years^7,8^. Other nematicides include non-fumigant nematicides with most being registered over 50 years ago, including the carbamate, oxamyl, and the organophosphate fenamiphos^8^. Both organophosphates and carbamates act as acetylcholinesterase inhibitors; their toxicity is not limited to nematodes and can be hazardous to humans and insects^9^. This potential for human toxicity has led to restrictions on the use of many carbamate and organophosphate pesticides.

Since the late 2000s, there has been an expansion of new nematicides on the market that have reduced user warning labels and specifically target PPN^8^. These newly-developed nematicides include fluopyram, fluensulfone, and fluazaindolizine. Unique to these three compounds’ structure is a trifluoro group, although other properties such as soil half-life and toxicity vary greatly^8^. Developing a greater understanding of how these compounds impact *M. incognita* is of particular importance as almost half of the $1 billion global nematicide market is used to control *Meloidogyne* spp.^4^. Both fluensulfone and fluazaindolizine have no defined mode-of-action. Fluazaindolizine is toxic to *Meloidogyne* species, but not to other species of PPN or free-living nematodes, indicating it may target only a fraction of PPN^10,11^.

Although other forms of nematode control exist such as plant resistance, soil solarization, cultural practices, and crop rotation, many of these methods take years for development, are limited in their effectiveness, or require specific knowledge about nematode biology and host preferences^7^. Chemical controls are the most reliable for growers facing challenges from PPN. As long relied on controls like broad-spectrum fumigants are being phased out, there is a concern that limited controls are available to replace them. This research aims to mediate these concerns by gaining a better understanding of how newly available nematicides interact with nematodes and the biological responses that occur when nematodes are exposed to these chemicals. This is critical for better stewardship of environmental inputs and development of these compounds to combat potential PPN resistance in the future. Therefore, the first objective of this study was to gain a general understanding of what genes are up- or down-regulated in response to nematicide exposure and compare this response across nematicides; possibly identifying a mode-of-action for fluazaindolizine and fluensulfone. The second objective was to examine how nematode detoxification gene expression and expression of oxidative stress response transcription factors were altered in response to nematicide exposure. Finally, the third objective was to determine commonly differentially expressed (DE) genes across compounds and the impact of these compounds on genes that encode fatty acid and retinoid-binding (FAR) proteins. FAR proteins are unique to nematodes and have shown to play an important role in nematode development and parasitism^12^. These common DE genes have the potential to be used to develop a chemical stress response RT-qPCR assay that could be used to quickly evaluate responses to novel nematicides and as a tool for making field management decisions in the future. This type of RT-qPCR assay would be extremely useful in evaluation of biological and traditional synthesized nematicides. It could result in a high-throughput system to evaluate potential nematicides versus timely plant assay systems traditionally used in nematicide discovery^4,8^.

## Results and discussion

**Figure 1:**
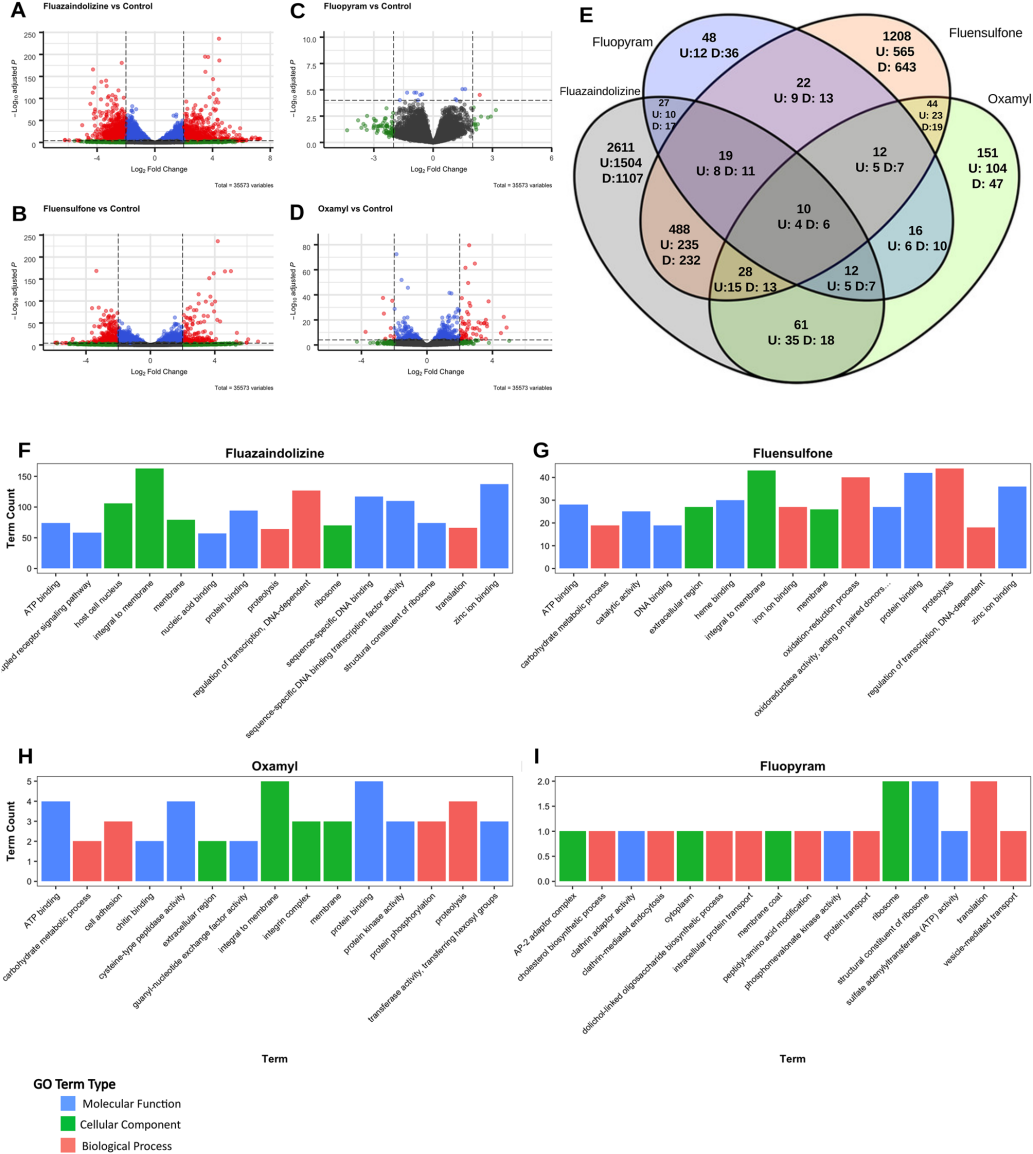
Summary of differentially expressed genes and the top Gene Ontology (GO) terms associated with significantly differentially expressed genes. *Meloidogyne incognita* second-stage juveniles were exposed to fluazaindolizine, fluensulfone, fluopyram, and oxamyl for 24-h and high throughput sequencing was used to determine gene expression compared to water control (N = 4 replicates/treatment). Volcano plots showing the differentially expressed genes in each treatment of this study (**A**–**D**). Dashed lines represent Log_2_ Fold Change values of − 2 and 2 and the y-axis the − Log_10_ of the adjusted *p*-value. The Venn diagram (**E**) shows the number of overlapping significantly (*p*-adjusted value < 0.05) differentially expressed genes between treatments. The number of up- or down-regulated genes, respectively (U and D). The top 15 Gene Ontology (GO) were examined in each treatment (**F**–**I**). The number of significantly (*p*-adjusted value < 0.05) differentially expressed genes in a treatment per a GO term are represented in the bar graphs. Color in each graph indicates the broader GO category each GO term belongs to.

*Meloidogyne incognita* gene expression was influenced the most by fluensulfone and fluazaindolizine, with 1208 and 2611 significantly DE genes, respectively (Fig. 1A,B,E). Oxamyl and fluopyram resulted in a much smaller transcriptomic response, with only 151 and 48 significantly DE genes, respectively (Fig. 1C–E). To gain a sense of the functionality of these DE genes, the top 15 GO (Gene Ontology) terms associated with DE genes for each nematicide were examined (Fig. 1F–I). There were no common GO terms across all nematicides, but in fluazaindolizine and fluensulfone, DE genes had clear activation of a transcriptional and translational responses occurring in the cell. Both nematicides shared GO terms like protein binding, regulation of transcription (DNA-dependent), and ATP binding (Fig. 1F,G). This clear transcriptional response was even more apparent in fluazaindolizine where greater than 100 of the DE genes had the associated GO term of sequence-specific DNA binding transcription factor activity (GO:0003700), sequence-specific DNA binding (GO:0043565), regulation of transcription, DNA-dependent (GO:0006355), and ribosome (GO: 0005840). In *M. incognita* treated with oxamyl, it was clear that membrane and cuticle modification may be the primary reaction to this nematicide. More than 3 DE genes belonged to each GO term involved in protein modification (protein phosphorylation GO:0006468; protein binding GO:0005515, proteolysis GO:0006508, protein kinase activity GO:0004672, ATP binding GO:0005524) and membrane components (integral to the membrane GO:0016021, membrane GO:0016020) (Fig. 1H). There were > 2 DE genes associated with chitin binding (GO:0008061), cell adhesion (GO:0007155), and integrin complex (GO:0008305). This could indicate that DE genes are acting to modify or reinforce the nematode cuticle. *Meloidogyne incognita* treated with fluopyram had relatively few DE genes to examine for GO term trends. However, the DE genes fell into GO term categories associated with endocytosis and cellular transport (AP-2 adapter complex GO:0030122, clathrin adaptor activity GO: 0035615, clathrin-mediated endocytosis GO:0072583, intercellular protein transport GO:0010496, protein transport GO:0015031, vesicle-mediated transport GO:0016192) (Fig. 1I). The GO terms associated with DE genes in *M. incognita* exposed to fluopyram indicate that cells may be enclosing fluopyram in vesicles to prevent the toxic effects of the compound. This has been observed in other eukaryotes as a way to detoxify heavy metals and bacterial toxins^13,14^. Further exploration of vesicle transport, membrane and cuticle modifications in *M. incognita* exposed to nematicides could provide other areas of potential nematicide development and provide more information on how *M. incognita* may develop tolerance or resistance to nematicides in the future (Table 1).

**Table 1:** Key findings and commercial nematicide product information.

Active ingredient	Commercial name	Available for use (release date)^a^	Mode-of-action^a^	Key findings from this study
Fluazaindolizine	Salibro™/Reklemel™	Under registration	Unknown	Strongest impact on nematode gene expression Mixed impact on cellular detoxification but generally promoted expression of detox genes Largest negative effect on genes that are involved in nutrient scavenging from the environment
Fluensulfone	Nimitz^®^	Available (2014)	Unknown	2nd largest alteration to nematode gene expression Shared the most affected genes with Fluazaindolizine Mixed influence on cellular detoxification, generally negative
Fluopyram	Velum Prime^®^/Indemnify^®^	Available (2010)	Succinate dehydrogenase inhibitor	Smallest impact on nematode gene expression Did not alter cellular detoxification
Oxamyl	Vydate L, C-LV^®^	Available (1972)	Acetylcholinesterase inhibitors	Negative impact on cellular detoxification Weak effect on nematode gene expression

^a^Information obtained from Desaeger et al.^8^.

### Nematode xenobiotic detoxification

Knowledge of xenobiotic detoxification in nematodes has mostly been explored in the model organism *Caenorhabditis elegans.* In *C. elegans,* detoxification is a two-step consecutive process. In step 1, functional groups are added, such as hydroxyl groups, to the xenobiotic to increase polarity and solubility of the compound^15^. These functional groups can be, but are not always, required for the second step. In step 2, the xenobiotic is further catalyzed to promote solubility and eventual excretion from the cell^15^. Cytochrome p450s (CYP) are an important class of enzymes involved in step 1. Step 2 enzyme classes include UDP-glucuronosyl transferases (UGTs) and glutathione S-transferases (GSTs). Final excretion of xenobiotics after step 2 modifications is done by ATP-binding cassette transporters (ABCs)^15^. All individual *M. incognita* genes involved in xenobiotic detoxification and their corresponding expression levels in this experiment can be found in Supplemental Fig. S1.

**Figure 2:**
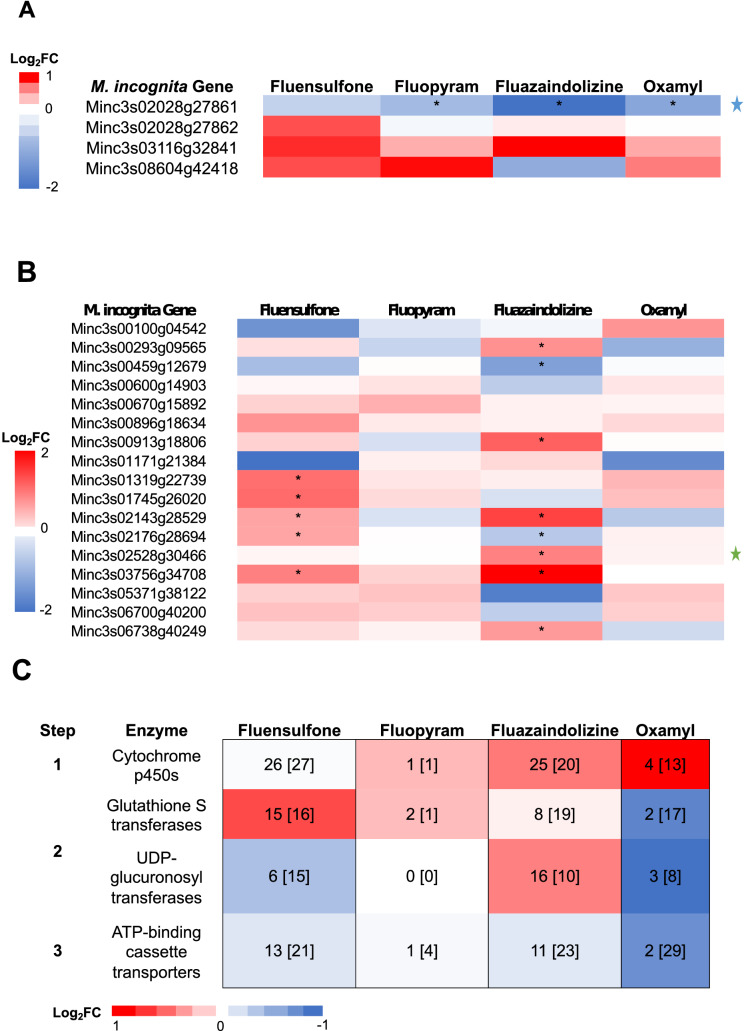
Gene expression of transcription factors and enzymes involved with nematode xenobiotic detoxification. *Meloidogyne incognita* second-stage juveniles were exposed to fluazaindolizine, fluensulfone, fluopyram, and oxamyl for 24-h and high throughput sequencing used to determine gene expression compared to water control (N = 4 replicates/treatment). In each panel expression in the form of Log_2_ Fold Change (Log_2_FC) red indicates up-regulated expression compared to control, and blue indicates down-regulation. Expression of transcription factors in the *Miskn1-like* family (**A**) and *Midaf16-like* family (**B**) are shown with colored stars representing genes of interest (blue star—*Miskn1-like-1* and green star—*Midaf16-like-1*). Average expression of differentially expressed genes at each step of nematode xenobiotic detoxification (**C**). The number significant up-regulated and down-regulated genes at each step are shown in the cells of panel (**C**), with down-regulated genes in the brackets. Asterisks throughout the figure represent significantly differentially expressed genes (*p*-adjusted value < 0.05).

Two known transcription factor families that play a role in regulating stress responses, including xenobiotic detoxification in *M. incognita*, are *Miskn1-like* and *Midaf16-like,* which control the expression of between 500 to 846 genes^16,17^. *Miskn1-like* and *Midaf16-like* are orthologous to the *C. elegans* genes *skn-1* and *daf-16* and were up-regulated when *M. incognita* was exposed to hydrogen peroxide, an oxidative stressor^16^*.* There are four *Miskn1-like* genes in *M. incognita*. *Miskn1-like-1* was the only ortholog significantly expressed in this study (Fig. 2A). This gene (Minc3s02028g27861) was down-regulated by fluopyram, fluazaindolizine, and oxamyl between 52 and 80% (Fig. 2A). Basso et al.^16^ found that a knock out of the *Skn-1* ortholog, *MiSkn1-like1,* resulted in the down-regulation of *MiGst1-like1*, a GST ortholog by 80%.

In this study, there were 17 *Daf-16-like* proteins found to be expressed in *M. incognita* across all nematicides, however, they were only differentially expressed in the fluensulfone and fluazaindolizine treatments (*p*-adjusted value < 0.05) (Fig. 2B). When exposed to fluensulfone, five of the *Daf-16-like* genes were up-regulated, with expression increase ranging from 1.6- to 2.2-fold. After exposed to fluazaindolizine, six *Daf-16* like genes were only up regulated between 1.8- and 4.0-fold, and two genes were down-regulated between 20 and 30% (Fig. 2B). Basso et al.^16^ found that *MiDaf16-like1* was up-regulated under conditions of oxidative stress, during plant parasitism, and early stages of nematode development (J2/third-stage juvenile). Knockout of *MiDaf16-like1* resulted in down-regulation of important components of antioxidant and detoxification pathways (peroxiredoxin, GSTs, peroxidase)^16^. Although *MiDaf16-like1* was not up-regulated after exposure of *M. incognita* to fluensulfone, 5 other *Daf-16* genes were (Fig. 2B). Fluazaindolizine also caused the up-regulation of 5 other *Daf-16* genes, 2 of which were also up-regulated by fluensulfone (Fig. 2B). *Meloidogyne incognita* exposed to fluazaindolizine and fluensulfone may be strongly upregulating *Daf16-like* genes to compensate for the down-regulation of *MiSkn1-like1* so that detoxification and stress response pathways can still be activated in the cell. The weak response of *Daf16-like* genes by fluopyram and oxamyl may be due to how the cell perceives these compounds, their overwhelming toxicity, or because of their modes-of-action.

All of the nematicides used in this study have been shown in in vitro assays to have varying levels of toxicity to *M. incognita*^18^. Fluazaindolizine is a slower acting nematicide, with a 24-h EC_50_ approximately 2 × that of fluensulfone and oxamyl, and 200 × that of fluopyram^18^. However, in this study, toxicity did not correlate with a strong up-regulation across cytochrome p450s. There were 128 genes identified with Pfam CYP domains in the *M. incognita* genome^19,20^, 103 of which were found to have expression data in at least one of the four nematicides evaluated. Of the 103 expressed genes with CYP domains, most were DE when *M. incognita* was exposed to fluensulfone and fluazaindolizine, 53 and 45, respectively. Only 2 genes were DE by fluopyram and 17 by oxamyl. On average CYPs were up-regulated across three nematicides, the average (arithmetic mean) fold-change of DE genes was 1.1-, 1.2-, and 1.5-fold up regulated by fluopyram, fluazaindolizine, and oxamyl, respectively (Fig. 2C). Exposure of *M. incognita* to fluensulfone resulted in an average reduction in expression of CYPs by ~ 2% (Fig. 2C). Overall, each nematicide tested had a unique expression pattern of CYPs, with little overlap, indicating that particular CYPs may be activated by different types of xenobiotics. This is the case in other systems. *Chironomus riparius*, an aquatic fly, showed twofold up-regulation of *CrCYP4G* after exposure to the biocide tributyltin but, after exposure to endocrine disrupters nonylphenol and bisphenol A, expression of *CrCYP4G* was only half that of the control^21^. In mice, multiple CYPs in the same family had both strong up-regulation and down-regulation in response to acrylamide^22^. Lewis et al.^23^ also demonstrated a differential regulation response in CYPs in *C. elegans* after exposure to two different organophosphates.

Only two CYP genes were differently expressed across all four nematicides. The first, Minc3s00305g09802, a *C. elegans CYP*-25A family ortholog, was down regulated with reductions in expression of 1.4-, 1.5-, 2-, and 3.3-fold for fluopyram, oxamyl, fluazaindolizine, and fluensulfone, respectively. Lewis et al.^23^ exposed *C. elegans* to two different organophosphates, dichlorvos and fenamiphos, which caused 2.4- and 1.7-fold increases in expression of *cyp-25A6*, respectively. In *M. incognita*, this CYP may not be needed to detoxify the nematicides tested in this study, hence the down-regulation observed across all nematicides. However, further research is warranted on the functionality of Minc3s00305g09802 due to its common response across all nematicides with different modes-of-action.

The second CYP DE gene common to all nematicides, Minc3s00532g13848, a *cyp-13A11* and *cyp-13A12 C. elegans* ortholog, was up-regulated with an increase in expression of 1.7-, 1.8-, 16.7-, and 32-fold, for fluopyram, oxamyl, fluazaindolizine, and fluensulfone, respectively. In *C. elegans, cyp-13A11* was up-regulated in response to a variety of environmental conditions. This included exposure to polychlorinated biphenyls (ubiquitous organic chlorine containing chemicals) where *cyp-13A11* was up-regulated 2 to 4-fold after exposure^24^. Under extreme acidic conditions, pH < 3, *cyp-13A11* was 5-fold up-regulated in *C. elegans*^25^. *Caenorhabditis elegans cyp-13A11* also plays an important role in nematode longevity as a part of a stress resistance response pathway^26^. If the function of Minc3s00532g13848 in *M. incognita* is similar to that of *cyp-13A11* in *C. elegans* it is not surprising that such an important stress response mediator was so highly up-regulated during nematicide exposure.

One of the enzymes involved in the second step of cellular detoxification is glutathione S-transferases (GST). There were 59 genes found in *M. incognita* with GST Pfam identified domains, 58 of which were expressed in *M. incognita* in response to exposure to nematicides in this study^19,20^. However, there were no DE genes across all four nematicides. Fluensulfone and fluazaindolizine had 31 and 27 DE GSTs, with 16 and 19 down-regulated, respectively. Oxamyl-treated *M. incognita* had 17 GSTs that were down-regulated and 2 that were up-regulated genes. The average (arithmetic mean) expression of these DE genes was 1.3-, 1.1-, and 1.0-fold up regulated by fluensulfone, fluopyram, and fluazaindolizine, respectively (Fig. 2C). By contrast, *M. incognita* exposed to oxamyl had 45% reduction in expression on average of DE genes (Fig. 2C). The only common DE genes across fluensulfone, fluazaindolizine, and oxamyl were Minc3s03593g34266, Minc3s00012g00790, and Minc3s00365g11065, which were down-regulated between 10 and 76% by all three nematicides. Minc3s03593g34266 and Minc3s00365g11065, are orthologs to the same 3 *C. elegans* GSTs (*gst-6*, *gst-33*, and *gst-13*). When exposed to cinnamaldehyde for just 4-h, *gst*-6 and 11 other GSTs were up-regulated in *C. elegans*^27^. A 72-h exposure to tris(1,3-dichloro-2-propyl), phosphate 10 GSTs were up-regulated including *gst-6* and *gst-33* in *C. elegans*^28^. Although the opposite trend was observed in this study for the *gst-6* and *gst-33* orthologs in *M. incognita*, the overall trend was up-regulation of GSTs by fluensulfone and fluazaindolizine. The down-regulation of GSTs by oxamyl was not unexpected, as there was significant down-regulation of *MiSkn1-like1* and no significant change in expression of *Daf-16-like* genes, two know regulators of GST expression^16,29^.

The other second step enzymes are UDP-glucuronosyl transferases (UGTs). In *M. incognita* there were 92 genes that contained a UGT domain, 65 of which were expressed in at least one of the nematicides^19,20^. Similar to GSTs, fluazaindolizine and fluensulfone had the most DE UGTs, 26 and 21, respectively (Fig. 2C). After exposure to oxamyl only 11 UGTs were DE and none were DE after exposure to fluopyram (Fig. 2C). The average (arithmetic mean) expression of DE UGTs was 24 and 50% reduced by fluensulfone and oxamyl, respectively (Fig. 2C). However, when *M. incognita* was treated with fluazaindolizine, the average (arithmetic mean) expression of DE UGTs was 1.2-fold increased. Minc3s10765g44349, 95% down-regulated by fluazaindolizine was orthologous to *C. elegans* gene *ugt-48.* In contrast to our results, in *C. elegans ugt-48* was up-regulated 1.75-fold in response to 500 mg/L dose of acrylamide, a known neurotoxin^30^. Oxamyl, a neurotoxin, did not have a significant impact on expression of Minc3s10765g44349 in our study. Minc3s01959g27433, which is an ortholog to *C. elegans ugt-60*, was significantly down-regulated by 70 and 65% after exposure to fluensulfone and fluazaindolizine, respectively. Expression of the *ugt-60* ortholog in *Bursaphelenchus xylophilus* parasitizing *Pinus*, was increased eightfold potentially as a response to plant defenses, such as reactive oxygen species and toxic secondary metabolites^17,31^. This particular UGT may only be responsive to oxidative stress and may not be needed to detoxify the nematicides fluensulfone and fluazaindolizine.

The final step in cellular detoxification is export from the cell by ATP-binding cassette transporters (ABCs). In the *M. incognita* genome there are 100 genes with an ABC domain, 75 of which were expressed across all four nematicides considered in this study^19,20^. Unlike with the other steps of detoxification, ATP-binding cassette transporters were on average down-regulated across the nematicides. Greater than 30 different ABCs were DE in the fluensulfone, fluazaindolizine, and oxamyl treatments, but only 5 in the fluopyram treatment. The average (arithmetic mean) expression of these genes was reduced 11, 3, 10 and 40%, for fluensulfone, fluopyram, fluazaindolizine and oxamyl, respectively (Fig. 2C). Only 2 genes were differentially expressed after exposure of *M. incognita* to all nematicides. The first was Minc3s02840g31763 which was down-regulated 33% by both fluensulfone and fluazaindolizine, and 53% and 40% by fluopyram and oxamyl, respectively. The other common DE ABC was Minc3s01359g23118, which was also down-regulated across all nematicides between 35–77%, with the highest down-regulation by oxamyl and fluensulfone.

In this study, each nematicide had its own pattern of up- and down-regulated ABCs, similar to that seen in CYPs. This selective up- or down-regulation has been observed in other systems. ABC transporter expression pattern varied greatly across different ABC genes in the fungus *Botrytis cinerea* and insect *Helicoverpa armigera* after exposure to a variety of plant defense compounds and various insecticides, respectively^32,33^. In *C. elegans*, ABC transporters played an important role in conferring resistance to ivermectin, a neurotoxin. In ivermectin resistant *C. elegans*, 9 different ABC transporters are up-regulated compared to wild-type *C. elegans* between 1.2- to 5-fold^34^. Yan et al.^34^ found that *mrp-1* and *pgp-2* were the most important ABCs for conferring ivermectin resistance in *C. elegans*. Knockout mutants of *mrp-1* and *pgp-2* had reduced egg production, motility, and pharyngeal pumping after ivermectin exposure. This contrasts with our results, where orthologs of *mrp-1* and *pgp-2* (Minc3s00470g12880, Minc3s00678g15963, Minc3s00849g18066, and Minc3s06399g39771) were significantly down-regulated in *M. incognita* treated with fluensulfone, oxamyl, and fluazaindolizine. While this overall down-regulation of ABCs is positive, indicating *M. incognita* is still susceptible to the toxic effects of these nematicides, it is worrisome that the up-regulation of ABC genes in other nematodes has been linked to resistance to nematicides^34^. Examining how gene expression across the detoxification pathway changes in *M. incognita* after repeated exposures to nematicides would an important area of research to pursue as this could be an avenue for potential resistance development in *M. incognita* to fluopyram, oxamyl, fluensulfone, and fluazaindolizine.

Expression of Minc3s02028g27861, Minc3s00305g09802, Minc3s00532g13848, and Minc3s06909g40472 were validated in a repeated nematicide exposure experiment using RT-qPCR. Minc3s00175g06781 expression was validated using RNA from the original experiment in RT-qPCR. Mean fold-change of expression relative to actin in each nematicide treatment followed the same directionality and approximate magnitude observed in the RNAseq experiment for each of the genes evaluated. The relative expression data and Spearman's rank correlation coefficient can be found in Supplemental Table S2.

### Common differentially expressed genes across nematicides

**Figure 3:**
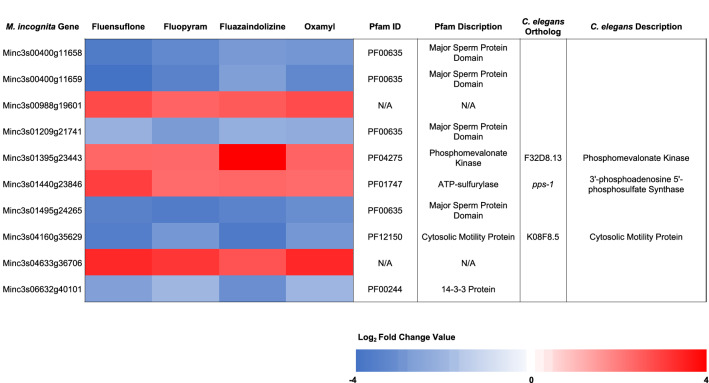
Common differentially expressed genes across treatments. *Meloidogyne incognita* second-stage juveniles were exposed to fluazaindolizine, fluensulfone, fluopyram, and oxamyl for 24-h and high throughput sequencing used to determine gene expression compared to water control (N = 4 replicates/treatment). The heatmap shows expression of genes that were common to four nematicides with *p*-adjusted value < 0.05 and a Log_2_ Fold Change (Log_2_FC) of > 2 or < − 2. Red indicates up-regulated expression compared to control, and blue indicates down-regulation. Pfam ID indicate the Pfam identity of the domain found in the gene and the description of that domain. If the gene had an ortholog to a *Caenorhabditis elegans* the gene name and description are provided in the figure.

There were only 10 significant (*p*-adjusted value < 0.05) common DE genes found after *M. incognita* J2 were exposed to oxamyl, fluensulfone, fluopyram, and fluazaindolizine (Fig. 3). These genes also had a Log_2_FC value that were > 2 or < − 2. Of these, only three had orthologs in *C. elegans*, Minc3s01395g23443, Minc3s01440g23846, and Minc3s04160g35629. The gene Minc3s01395g23443, an ortholog of the *C. elegans* gene F32D8.13, encodes for a phosphomevalonate kinase^17^. This gene was 4-fold up-regulated by fluopyram, fluensulfone, and oxamyl, and 11-fold up-regulated by fluazaindolizine (Fig. 3). Phosphomevalonate kinase is a critical enzyme in the mevalonate pathway, which is responsible for synthesizing cholesterol, along with other molecules like ubiquinone, coenzyme Q, dolichols, and isoprenoids^35^. These molecules are essential in cell functions like membrane integrity, signaling, glycosylation, and energy homeostasis^35^. The mevalonate pathway is important for monitoring and responding to mitochondrial impairment in *C. elegans*^36^. Fluopyram acts as a succinate dehydrogenase inhibitor in fungi and impacts the functionality of the mitochondria^37,38^, therefore upregulating components of the mevalonate pathway could help the cell to overcome this distress.

Minc3s01440g23846 was another common DE gene which is orthologous to the *C. elegans* gene *pps-1*, a 3′-phosphoadenosine 5′-phosphosulfate (PAPS) synthase^19,39^. Expression of Minc3s01440g23846 was 4-fold higher than the water control when *M. incognita* was treated with fluopyram, fluazaindolizine, and oxamyl, and 6-fold higher when treated with fluensulfone (Fig. 3). PAPS synthases generate the activated sulfate donor required for all sulfation reactions in eukaryotes, and their supply can be rate-limiting^42^. These sulfation reactions are important in extracellular protein modification and components of extracellular matrices^42^. In *C. elegans*, *pps-1* knock-outs resulted in a lethal phenotype, larvae only survived 1–2 days and had abnormal hypodermal cell shapes and unusual position of muscle cells on the nematode body^43^. Along with their central role in nematode development, PAPS synthases are also important for xenobiotic metabolism. Sulfotransferases catalyze the transfer of a sulfonate group from PAPS to xenobiotics, which will often inactivate them^44^. The up-regulation of the essential co-factor for sulfonation (PAPS), appears to be a common way for *M. incognita* to combat nematicide toxicity, regardless of mode-of-action. In this study, the two *C. elegans* orthologs to the sulfotransferase, *ssu-1* (Minc3s00449g12507 and Minc3s00013g00810)^17,39^ were up-regulated > 3.5-fold by fluazaindolizine (*p*-adjusted value < 3.68e−6), but expression was unaffected by fluensulfone, fluopyram, and oxamyl. Sulfonation may play an important role in detoxification of fluazaindolizine due to the high up-regulation of PAPS synthases and sulfotransferases themselves.

The final common DE gene with a *C. elegans* ortholog was Minc3s04160g35629, which is orthologous to the gene K08F8.5^39^. Expression of Minc3s04160g35629 was reduced > 92% when *M. incognita* were exposed to fluazaindolizine and fluensulfone and ~ 87% when exposed to oxamyl and fluopyram (Fig. 3). K08F8.5 has no described function, but contains two cytosolic motility protein domains^19^. Expression of this gene was enriched in male fourth-stage larvae of *C. elegans*^19,40^.

Of the 10 common DE genes, four were identified to have major sperm protein (MSP) domains (Minc3s00400g11658, Minc3s00400g11659, Minc3s01209g21741, Minc3s01495g24265) (Fig. 3)^19^. All four genes were down-regulated in *M. incognita* with expression reduction ranging from 79–94% (*p*-adjusted < 0.05) (Fig. 3). Major sperm proteins behave like actin and facilitate the crawling movement of nematode sperm^41^. All four genes were down-regulated across treatments. MSP can associate with cytosolic motility proteins to enhance or reduce their filamentation^41^. MSP has been shown to have extracellular functions by simulating oocycte maturation in *C. elegans*^42^. The down-regulation of these MSP genes and Minc3s04160g35629 after nematicide exposure may function in slowing nematode maturation until the nematode is out of a toxic environment. These proteins could also have additional functions outside of reproduction that help nematodes adapt to xenobiotic exposure. This apparent down-regulation could also be a biproduct of up-regulation of these genes in the water control.

Minc3s06632g40101 was also highly down-regulated across the nematicides ranging from 10–23% reduction in expression (Fig. 3). While Minc3s06632g40101 does not have a *C. elegans* ortholog, the Pfam identity is a 14-3-3 protein^19^. These evolutionarily conserved phosphorylation binding proteins that contain a 14-3-3 domain are regulators of a variety of cellular processes, including apoptosis, DNA repair, cell cycle progression, and reproduction^43,44^. In *C. elegans,* two 14-3-3 proteins have been shown to help regulate nematode longevity^45^ and regulate the *daf-2*/insulin-like pathway, which is responsible for stress resistance^46^. The gene responsible for regulating the *daf-2*/insulin-like pathway in *C. elegans* is *ftt-2*, which binds DAF-16 and prevents its localization to the nucleus where it regulates transcription^46^. If Minc3s06632g40101 does function like *fft-2*, it is unsurprising this gene was down-regulated in all the treatments as it would be an important step in starting the detoxification pathway cascade. The true functionality of this particular 14-3-3 protein (Minc3s06632g40101) in *M. incognita* is undetermined^19,39^. Research expanding the understanding of the functionality and binding specificity of Minc3s06632g40101 would be beneficial in understanding the role it plays in nematode response to nematicide exposure.

The final two common DE genes in this study, Minc3s00988g19601 and Minc3s04633g36706, at this time have no identified Pfam domains or known *C. elegans* orthologs so their function remains unknown^19^. However, their expression was highly expressed across all treatments with up-regulation ranging from 4.5- to 7.9-fold (Fig. 3). Minc3s00988g19601 has four orthologs, two each in *M. javanica* and *M. arenaria*^19^. Minc3s04633g36706 has one ortholog each in *M. javanica* and *M. graminicola*, and two orthologs in *M. arenaria*. However, no functionality or conserved Pfam domain have been described for these proteins in any species. Future research should be devoted to understanding functionality of these genes as they appear unique to the *Meloidogyne* spp. and are highly expressed in *M. incognita* when exposed to a range of nematicides (Fig. 3.)

### Nematode fatty acid retinoid binding proteins

**Figure 4:**
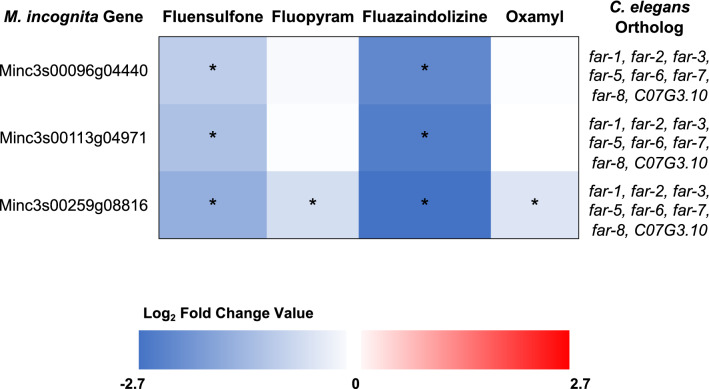
Expression of fatty acid and retinoid binding (FAR) genes. *Meloidogyne incognita* second-stage juveniles were exposed to fluazaindolizine, fluensulfone, fluopyram, and oxamyl for 24-h and high throughput sequencing used to determine gene expression compared to water control (N = 4 replicates/treatment). The heatmap shows the expression of the 3 genes in *M. incognita* that contained a Pfam domain for FAR. Expression is in the form of Log_2_ Fold Change (Log_2_FC) with red indicating up-regulated expression compared to control, and blue indicating down-regulation. Asterisks throughout the figure represent significantly differentially expressed genes (*p-*adjusted value < 0.05). If the gene had orthologs in *Caenorhabditis elegans*, the gene name is indicated.

Common among treatments was the down regulation of fatty acid retinol binding (FAR) proteins. There are three genes in *M. incognita* that have FAR domains (Minc3s00096g04440, Minc3s00113g04971, Minc3s00259g08816)^19^. In this study, all three proteins were down-regulated by fluensulfone and fluazaindolizine (Fig. 4). When *M. incognita* was treated with fluazaindolizine, expression of all 3 genes was reduced 80–85%, but was only reduced 52–68% when treated with fluensulfone (Fig. 4). Minc3s00259g08816 was the only gene that was significantly down-regulated by fluopyram and oxamyl, with a reduction in expression of 40% and 35%, respectively (Fig. 4).

FAR proteins help to scavenge fatty acids and retinoids from the environment, transport signaling molecules throughout the nematode pseudocoelom, including developmental signal molecules, and are important for glycoprotein synthesis^12,47,48^. In *Globodera pallida*, *Gp-FAR-1* bound to precursors of plant defense molecules, including jasmonic acid signaling pathway, and inhibited their mortification in vitro^49^. The silencing of a FAR protein in *Pratylenchus penetrans*, *Pp-far-1,* reduced reproduction on soybean hairy root lines between 44 and 70%^12^. Tomato roots with over expression of *M. javanica Mj-far-1* and then inoculated with *M. javanica* had larger gall size than roots not expressing *Mj-far-1*^50^*.* In this same study, tomato genes involved in biotic stress, cell wall development, and defense related secondary metabolite precursors were all differently expressed in response to *Mj-far-1.* Fluazaindolizine and fluensulfone have shown to be strong nematicides with the ability to suppress *Meloidogyne* spp. reproduction in both greenhouse studies and field trials on a variety of different hosts^10,18,51–54^. The strong down regulation of FAR proteins in fluazaindolizine and fluensulfone-treated *M. incognita* could contribute to the ability of these nematicides to suppress nematode infection and reproduction.

## Conclusion

This study revealed that when compared with exposure to oxamyl and fluopyram, *M. incognita* mounted a stronger transcriptional response after exposure to fluensulfone and fluazaindolizine, including in the three different stages of xenobiotic detoxification, where these, treatments elicited the strongest fold-changes in expression (Table 1). Oxamyl and fluopyram at doses examined in this study, did not stimulate strong changes in expression of nematode detoxification genes. However, further exploration and monitoring of how long-term, repeated exposure to nematicides changes expression of detoxification genes in *M. incognita* is warranted as it could evolve into an avenue of nematicide tolerance or resistance. In this study, the 10 commonly DE genes across the four treatments were also examined. Of the 10 genes, three were found that had potential functions related to detoxification, however the remaining seven had unknown functionality. These genes would be great candidates for further research, as they are strongly expressed across all the treatments and could provide further insight into how *M. incognita* responds to nematicide exposure. Finally, genes encoding FAR proteins were significantly down-regulated across all nematicides, but down-regulation was the strongest by fluazaindolizine. FAR proteins have been shown to play a crucial role in parasitism and nematode development and their down-regulation is a further way fluensulfone and fluazaindolizine may reduce nematode reproduction. Understanding how nematicides alter nematode biology beyond general toxicity is worth further exploration as a potential way to bolster less effective management strategies by amplifying their effects with lower chemical inputs. This study provided a basic understanding of the transcriptional response in *M. incognita* to nematicide exposure and data that can be used for future nematicide work.

## Materials and methods

### Nematode collection

*Meloidogyne incognita* originally collected from grape (*Vitis vinifera*) in Parlier, CA was established in culture by inoculating a single egg mass on tomato (*Solanum lycopersicum*) ‘Rutgers’. The identity of the *M. incognita* isolate was confirmed by molecular analysis by the North Carolina Department of Agriculture and Consumer Services (Raleigh, NC). After 6–8 weeks, additional tomato plants were inoculated with 12–15 egg masses hand-picked from the plant inoculated with a single egg mass. To obtain *M. incognita* second-stage juveniles (J2), egg masses were hand-picked from infected tomato roots and placed on a 1.5-cm diameter plastic hatching chamber with a 30 μm nylon mesh in a 40 mL beaker containing water^55,56^. Hatched *M. incognita* J2 were collected after 3 days and stored at 4 °C until use, no more than 2 days.

### Nematicide exposure

Previously established dose–response curves for *M. incognita* were used to determine nematicide doses used in this study^18^. Four nematicides, fluazaindolizine, fluensulfone, fluopyram, and oxamyl were evaluated at four doses, 208, 200, 2, and 63 ppm, respectively. All concentrations are of active ingredient, but formulated product was used for exposure. Doses represent ED_90_ calculated by the dose–response model previous developed^18^. Additionally, in Supplemental Table S3 the mean percentage active *M. incognita* J2 for each treatment is presented. In a 1.7 mL microcentrifuge tube, 5000 M*. incognita* J2 were suspended in 100 µL of water and treated with 900 µL of a nematicide solution to obtain the appropriate dose for each nematicide treatment; a water control was also included. Each treatment was replicated four times. Nematodes were incubated in the nematicide solution for 24-h at room temperature, after which nematodes were frozen in liquid nitrogen and stored at − 80 °C until RNA extraction.

### RNA extraction, library preparation, and sequencing

The RNeasy Minikit^®^ (QIAGEN; Hilden, Germany) was used to extract RNA from each sample. The RNeasy Minikit^®^ manufacturer instructions for RNA isolation from plant tissue were used with modifications to the cell lysis step. Manufacturer provided lysis solution in the recommended amount was added to each tube along with 1 mm glass beads (Sigma-Aldrich; Darmstadt, Germany). Each sample was beat in a BioSpec 3110BX Mini-BeadBeater (BioPointe Scientific; Claremont, CA, USA) for 90 s at speed 25 (800 oscillations/per min). After bead beating, 20 μL of proteinase K (20 mg/mL) (Ambion Inc.; Austin, TX, USA) was added to each sample and then samples were incubated at 56 °C for 15 min. After incubation, the next steps of the RNeasy Minikit^®^ were followed including a RNase-DNase (QIAGEN) on column treatment. After RNA extraction, samples were sent to Oregon State University Center for Genome Research and Biocomputing (Corvallis, OR, USA) for cDNA library preparation using NEBNext^®^ Ultra RNA Library Prep Kit (Illumina; San Diego, CA). The cDNA libraries from all treatment replicates were uniquely barcoded, pooled, and sequenced on one lane of the Illumina HiSeq 3000 Platform using 100 base pair single-end reads. All raw sequencing data can be found under the NCBI BioProject PRJNA818683.

### Bioinformatic workflow

To remove sequencing adaptors and reads with Phred scores < 20, Trim Galore!^57^ was used. Trimmed and filtered reads were aligned to the *M. incognita* genome v3 (NCBI BioProject PRJEB8714)^20^ and reads per gene were counted using STAR^58^. All differentially expressed gene analyses and visualizations were done using R^59^. The STAR generated count was then used as input for differential gene expression determination by DESeq2^60^. Significant differentially expressed genes were genes that had an adjusted *p*-value < 0.05 and a log_2_Fold-Change value ≥ 2 or ≤ − 2. Enriched Gene Ontology (GO) terms for each treatment differentially expressed gene set were determined using the R package topGO^61^.

To enable the exploration of expression of detoxification genes, WormBase ParaSite^19^ was used to download gene IDs and protein coding sequences identified in each Pfam domain family (ABC transporters, cytochrome p450s, UDP-glucuronosyl transferases and glutathione S-transferases). Sequences from each gene family were aligned using MUSCLE^62^. Approximately maximum-likelihood gene trees for each gene family were constructed using FastTree-2.1.10^63^ and visualized using ggtree^64^.

### Gene expression validation

To validate expression of genes found in the RNAseq analysis, two-step RT-qPCR was used. Total RNA from the original RNAseq experiment or RNA isolated as described above from *M. incognita* J2 under the same experimental conditions was used to synthesize cDNA with the Verso cDNA synthesis kit (Thermo Fisher Scientific, Waltham, MA), according to manufacturer’s instructions. The amount of input RNA was standardized across all samples before cDNA synthesis. Quantitative PCR was performed on an Applied Biosystems StepOnePlus™ Real-Time PCR System (Thermo Fisher Scientific) with reactions containing 200 nM of primers, the manufacturer recommended amount of GoTaq^®^ qPCR Master Mix (Promega, Madison, WI), and reference dye CXR. The following thermocycler conditions were used: 95 °C for 3 min, 40 cycles of 95 °C for 15 s and 63 °C for 1 min, followed by the establishment of a melting curve using the following program: 95 °C for 15 s, 63 °C for 1 min, a slow ramp from 61 °C to 95 °C, and 95 °C for 15 s. For each nematicide treatment and the water control, qPCR was performed on three biological replicates and three technical replicates.

Primers were designed for five genes using IDT primerQuest tool (Integrated DNA Technologies, Coralville, IA) for RT-qPCR primer design with the default parameters. The genes chosen for RT-qPCR are outlined in Supplemental Table S1. Protein coding sequences for each gene to be validated were downloaded from WormBase ParaSite^19^ and used as input for IDT primerQuest tool. In order to measure relative expression and compare expression results found in the RNAseq study, *M. incognita MiACT* (Minc3s00730g16611) was used as the constitutively expressed housekeeping gene^16^. Primers were designed for *MiACT* using the method described above. Relative expression was determined using the 2^−ΔΔCT^ method^65^ and paired *t* test with a Bonferroni correction was performed in R^59^ to establish statistical differences between treatments and the untreated control. To examine correlation between the mean fold-change found in the RNAseq experiment and RT-qPCR, the correlation coefficient was calculated in R^59^. Data is presented for gene expression validation in Supplemental Table S2.

All local, national, and international guidelines were adhered to in the production of this study. Any plant material that mentioned was used with permission.

## Supplementary Information

Supplementary Figure 1.

Supplementary Legends.

Supplementary Table 1.

Supplementary Tables.
