# Supplementary figures and images for: Transcriptional response of Meloidogyne incognita to non-fumigant nematicides
Source: Sci Rep. 2022 Jun 13;12:9814. doi: 10.1038/s41598-022-13815-9 (PMC9192767; doi:10.1038/s41598-022-13815-9)

**A**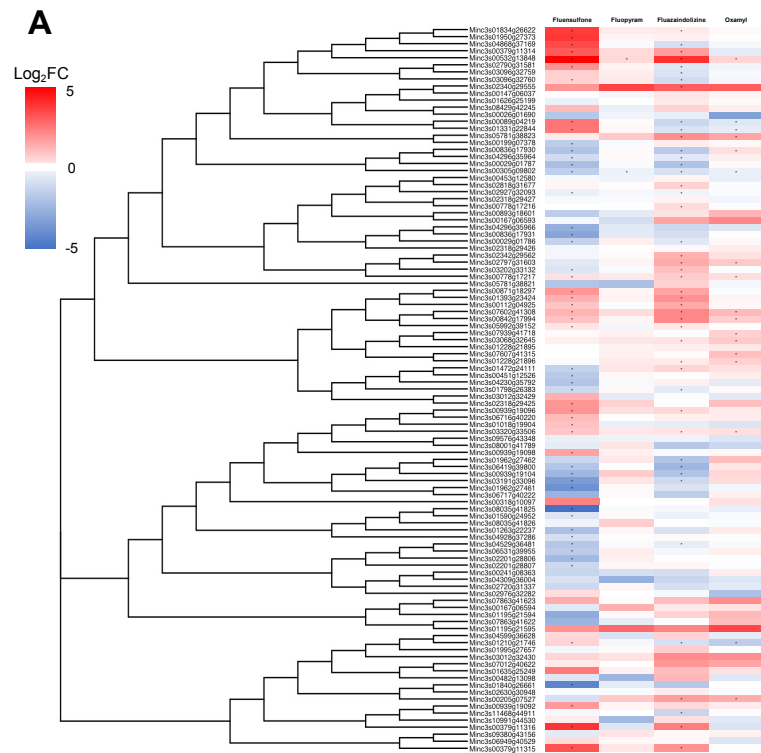**C**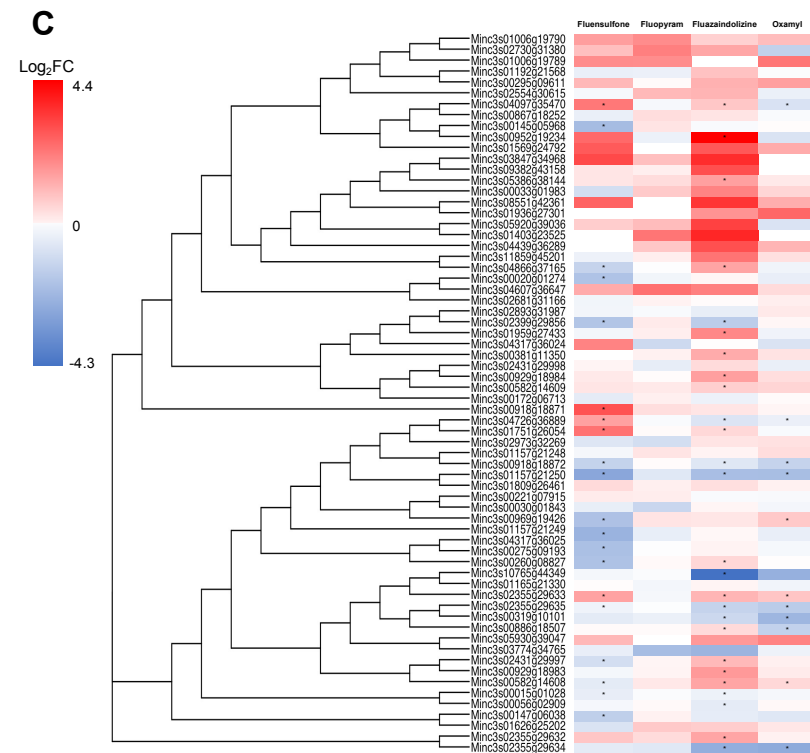**B**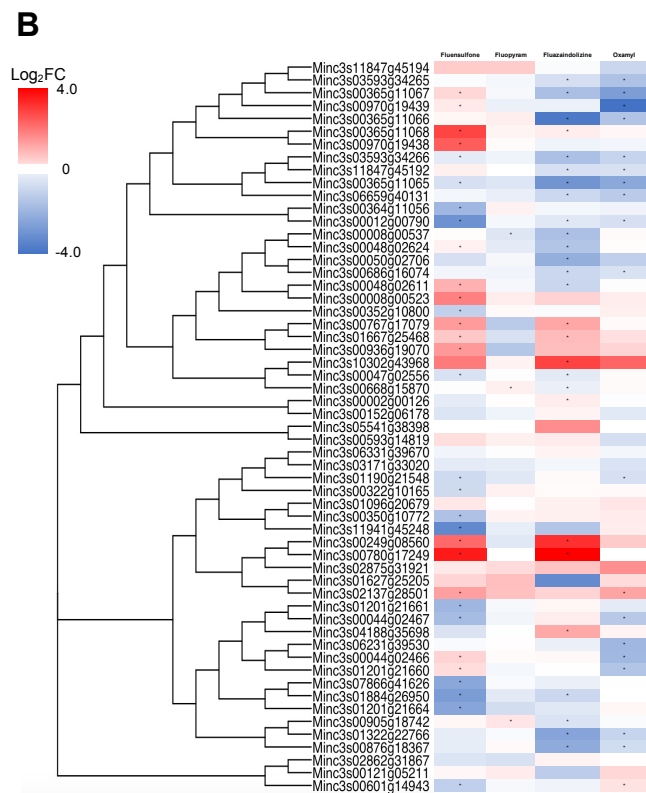**D**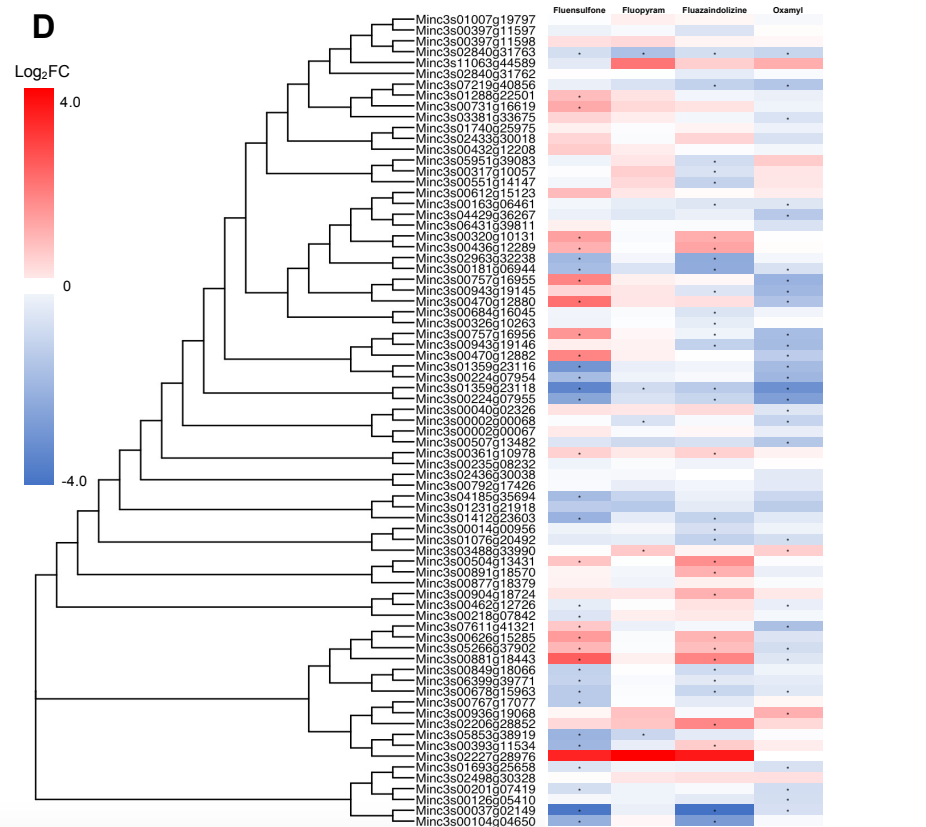

Supplement: Supplementary file 1 — Supplementary Figure 1. [file 41598_2022_13815_MOESM1_ESM.pdf]
