# Supplementary material for: Transcriptional response of Meloidogyne incognita to non-fumigant nematicides
Source: Sci Rep. 2022 Jun 13;12:9814. doi: 10.1038/s41598-022-13815-9 (PMC9192767; doi:10.1038/s41598-022-13815-9)
Supplement: Supplementary file 2 — Supplementary Legends. [file 41598_2022_13815_MOESM2_ESM.docx]

**Supplemental Figure 1.** Gene expression of nematode xenobiotic detoxification steps in *Meloidogyne incognita* after exposure to fluazaindolizine, fluensulfone, fluopyram, and oxamyl. *M. incognita* second-stage juveniles (J2) were exposed to nematicides for 24-hrs and high throughput sequencing used to determine gene expression compared to a water treated control (N=4 replicates/treatment). Expression of genes with Pfam domains of (A) cytochrome p450s, (B) glutathione S-transferases, (C) UDP-glucuronosyl transferases, and (D) ATP-binding cassette transporters were examined. Expression is in the form of Log_2_ Fold Change (Log_2_FC) with red colors indicating upregulated expression compared to control, and blue indicating downregulation. Asterisks throughout the figure represent significantly differentially expressed genes (*p* adjusted value < 0.05). If the gene had orthologs in *Caenorhabditis elegans,* the gene name is indicated. Phylogenetic trees indicate the level of similarity in sequence detoxification genes are to each other. Red and orange stars indicate CYPs of interest.
