# Supplementary material for: Transcriptional response of Meloidogyne incognita to non-fumigant nematicides
Source: Sci Rep. 2022 Jun 13;12:9814. doi: 10.1038/s41598-022-13815-9 (PMC9192767; doi:10.1038/s41598-022-13815-9)
Supplement: Supplementary file 3 — Supplementary Table 1. [file 41598_2022_13815_MOESM3_ESM.docx]

**Supplemental Table 1. RT-qPCR primer sequences for RNAseq Expression Validation**

| **Gene ID^a^** | **Pfam ID/gene ID^b^** | **Primer Name** | **Primer sequence (5’-3’)** | ***Tm*** | **GC%** | **Length (bp)^c^** |
| --- | --- | --- | --- | --- | --- | --- |
| **Minc3s02028g27861** | ***Miskn1-like-1* ^d^** | **27861-F** | **TCCAACCACCAACAGCAACA** | **59** | **50** | **84** |
|  |  | **27861-R** | **ACGTGAACGTTGCCTTGAATG** | **59** | **47.6** |  |
| **Minc3s06909g40472** | **Succinate Dehydrogenase cytochrome B small subunit** | **40472-4F** | **GTACTTTCGGCTGCTATTGTTATG** | **62** | **41.7** | **90** |
|  |  | **40472-4R** | **ACGTTCGCCAACGACTATT** | **62** | **47.4** |  |
| **Minc3s00532g13848** | **Cytochrome p450** | **13848-2F** | **GGGAGAAGATGCCGAAGAAT** | **62** | **50** | **96** |
|  |  | **13848-2R** | **GGTCCTCCTCCAAATGGATAAT** | **62** | **45.5** |  |
| **Minc3s00175g06781** | **Aconitase/3-isopropylmalate dehydratase large subunit, alpha/beta/alpha domain** | **06781-3F** | **GTACCGGAATGGGAACCATAA** | **62** | **47.6** | **106** |
|  |  | **06781-3R** | **CGACCAGTAGCCACAAGATAG** | **62** | **52.4** |  |
| **Minc3s00305g09802** | **Cytochrome p450** | **09802-5F** | **GTGGATCCGTTGACGTTCTTA** | **62** | **47.6** | **108** |
|  |  | **09802-5R** | **ACGTCTAACCAAATGAGCAATAAC** | **62** | **37.5** |  |
| **Minc3s00730g16611** | **Actin** | **actin3-1F** | **CCTCGACATCAAGGAGTTATGG** | **62** | **50** | **110** |
|  |  | **actin3-1R** | **CCGTGCTCAATCGGGTATTT** | **62** | **50** |  |

**^a^Gene ID from M. incognita genome v3 (NCBI BioProject PRJEB8714) ^b^Pfam domain associated with gene ^c^Length of qPCR product**

**^d^Primers from Basso et al. 2020 [16]**
