# Supplementary material for: Transcriptional response of Meloidogyne incognita to non-fumigant nematicides
Source: Sci Rep. 2022 Jun 13;12:9814. doi: 10.1038/s41598-022-13815-9 (PMC9192767; doi:10.1038/s41598-022-13815-9)
Supplement: Supplementary file 4 — Supplementary Tables. [file 41598_2022_13815_MOESM4_ESM.docx]

**Supplemental Table 2.** RT-qPCR validation of RNAseq results

|  |  | **Fold Change in Expression using RT-qPCR^c^** | | | |  |
| --- | --- | --- | --- | --- | --- | --- |
| **Gene ID^a^** | **Pfam ID/gene ID^b^** | **Fluensulfone** | **Fluopyram** | **Fluazaindolizine** | **Oxamyl** | **Correlation Coefficient ^d^** |
| Minc3s00305g09802 | Cytochrome p450 | 0.227* | 0.127* | 0.351* | 0.469* | 0.26 |
| Minc3s02028g27861 | *Miskn1-like-1* | 0.464* | 0.399* | 0.364* | 0.021* | 0.29 |
| Minc3s06909g40472 | Succinate Dehydrogenase cytochrome B small subunit | 2.400* | 3.493* | 3.977* | 2.431* | 0.60 |
| Minc3s00532g13848 | Cytochrome p450 | 14.251* | 2.143* | 11.545* | 1.841* | 0.96 |
| Minc3s00175g06781 | Aconitase/3-isopropylmalate dehydratase large subunit, alpha/beta/alpha domain | 0.654* | 0.386 | 0.574 | 0.952* | 0.29 |

**^a^**Gene ID from *M. incognita* genome v3 (NCBI BioProject PRJEB8714) **^b^**Pfam domain associated with gene or known gene name **^c^**Fold change in expression calculated relative to expression of *MiACT* (Minc3s00730g16611) using RT-qPCR and the 2–ΔΔCT method **^d^**For each gene, across all treatments, Spearman's rank correlation coefficient was calculated between the fold change in expression found using RT-qPCR and fold change found in the RNAseq experiment * Indicates mean fold-changes relative to actin in *M. incognita* treated with nematicide were significantly different from the water control (*p*-value < 0.05, Pair-wise T-test Bonferroni correction).

**Supplemental Table 3**. Average active *Meloidogyne incognita* second-stage juveniles (J2) in each nematicide treatment used for RNAseq experiment

| **Treatment** | **Concentration (ppm)^a^** | **Percentage Active J2^b^** |
| --- | --- | --- |
| Water Control |  | 90% |
| Fluopyram | 2 | 7% |
| Fluensulfone | 200 | 13% |
| Fluazaindolizine | 208 | 13% |
| Oxamyl | 63 | 2% |

**^a^**Concentration of active ingredient used for nematode exposure  **^b^**Each percentage active reflects the mean of four replicates where 100 nematodes/replicate were examined to count moving or not moving J2.
